# A Novel Bead-Based Immunoassay for the Measurement of Heat Shock Proteins 27 and 70

**DOI:** 10.3390/pathogens9110863

**Published:** 2020-10-22

**Authors:** Rose Njemini, Katrijn Verhaeghen, Tony Mets, Ilse Weets, Ivan Bautmans

**Affiliations:** 1 Frailty in Ageing Research Group, Vrije Universiteit Brussel, Laarbeeklaan 103, B-1090 Brussels, Belgium; Ivan.Bautmans@vub.be; 2 Gerontology Department, Vrije Universiteit Brussel, Laarbeeklaan 103, B-1090 Brussels, Belgium; 3 Laboratory of Clinical Chemistry and Radiology, Universitair Ziekenhuis Brussel, Laarbeeklaan 101, B-1090 Brussels, Belgium; katrijn.verhaeghen@uzbrussel.be (K.V.); iweets@vub.be (I.W.); 4 Department of Geriatric Medicine, Universitair Ziekenhuis Brussel, Laarbeeklaan 101, B-1090 Brussels, Belgium; Tony.Mets@vub.be

**Keywords:** heat shock proteins, single molecule assay, plasma, cell lysates

## Abstract

Heat shock proteins (HSPs) play an essential role in protecting proteins from denaturation and are implicated in diverse pathophysiological conditions like cardiovascular diseases, cancer, infections, and neurodegenerative diseases. Scientific evidence indicates that if HSP expression falls below a certain level, cells become sensitive to oxidative damage that accelerates protein aggregation diseases. On the other hand, persistently enhanced levels of HSP can lead to inflammatory and oncogenic changes. To date, although techniques for measuring HSPs exist, these assays are limited for use in specific sample types or are time consuming. Therefore, in the present study, we developed a single-molecule assay digital ELISA technology (Single Molecule Array—SIMOA) for the measurement of HSPs, which is time effective and can be adapted to measure multiple analytes simultaneously from a single sample. This technique combines two distinct HSP-specific antibodies that recognize different epitopes on the HSP molecule. A recombinant human HSP protein was used as the standard material. The assay performance characteristics were evaluated by repeated testing of samples spiked with HSP peptide at different levels. The limit of detection was 0.16 and 2 ng/mL for HSP27 and HSP70, respectively. The inter- and intra-assay coefficients of variation were less than 20% in all tested conditions for both HSPs. The HSP levels assayed after serial dilution of samples portrayed dilutional linearity (on average 109%, R^2^ = 0.998, *p* < 0.001, for HSP27 and 93%, R^2^ = 0.994, *p* < 0.001, for HSP70). A high linear response was also demonstrated with admixtures of plasma exhibiting relatively very low and high levels of HSP70 (R^2^ = 0.982, *p* < 0.001). Analyte spike recovery varied between 57% and 95%. Moreover, the relative HSP values obtained using Western blotting correlated significantly with HSP values obtained with the newly developed SIMOA assay (r = 0.815, *p* < 0.001 and r = 0.895, *p* < 0.001 for HSP70 and HSP27, respectively), indicating that our method is reliable. In conclusion, the assay demonstrates analytical performance for the accurate assessment of HSPs in various sample types and offers the advantage of a huge range of dilution linearity, indicating that samples with HSP concentration highly above the calibration range can be diluted into range without affecting the precision of the assay.

## 1. Introduction

Heat shock proteins (HSPs) are highly conserved proteins that are synthesized not only in response to cellular insults, but also under normal growth conditions in which cells have to handle the stress associated with activation, differentiation, and maturation [1]. They are classified into families according to their molecular weights—namely, the small HSPs (e.g., HSP27), HSP40, HSP60, HSP70, HSP90, HSP100, and HSP110; with some families having more than one member [2]. More recently, Kampinga and colleagues reported a new guideline for the classification of HSPs. This nomenclature comprises HSPB (small HSP), DNAJ (HSP40), HSPA (HSP70), HSPC (HSP90), HSPH (HSP110), and the human chaperonin families CCT (TRiC) and HSPD/E (HSP60/HSP10) [3]. A complete description of HSP nomenclature is beyond the scope of this paper (see Vos et al. [4] for an overview).

While the precise function of HSPs may vary according to specific HSP and depend on the context in which they are studied, all HSPs appear to mediate cellular protection in response to stressful situations [5,6,7,8]. HSPs display their cytoprotective properties, by directly interacting with various components of the tightly regulated programmed cell death machinery, upstream and downstream of the mitochondrial events [9]. At the pre-mitochondrial level, HSPs inhibit stress-activated kinases such as c-Jun N-terminal kinase and apoptosis signal regulating kinase 1 [10]. On the other hand, HSPs have been shown to bind pro-survival genes such as non-phosphorylated protein kinase C, AKT and BCL2, priming them for phosphorylation and protein stabilization [9]. HSPs prevent the translocation of BAX—a pro-apoptotic protein—from the cytoplasm to the mitochondria and inhibit apoptosis at the post-mitochondrial level by their suppressing effect on two pro-apoptotic molecules, Apaf-1 (apoptotic protease activation factor 1) and AIF (apoptosis inducing factor), implicated in the caspase independent and dependent pathways [11]. In a study on the use of hyperthermia as a co-adjuvant in cancer therapy, Sharma et al. [12] portrayed that Hep3B cells are more susceptible to death upon heat stress than HepG2 cells, and they postulated that the reason was due to a non-induction of HSP70 during heat stress in Hep3B cells. Moreover, the down-regulation of HSP70 by anti-sense constructs has been reported to have chemo-sensitizing properties [13]. Recently, Voss et al. reported that increased expression of HSP27 in primary human macrophages contributes to their prolonged survival [14]. HSP27 modulates the apoptotic cascade by sequestering cytochrome c, resulting in the inhibition of caspase-9, and by directly associating with caspase-3, inhibiting its activation. In addition, HSP27 can inhibit the Fas-induced apoptotic pathway by blocking the interaction of Daxx with Fas [15].

Enormous attention has focused on HSPs as molecular chaperones [6,9,16], and the interaction of HSPs with cellular components is thought to have therapeutic potentials [17,18,19]. In this perspective, HSPs have been implicated in diverse pathophysiological conditions like cardiovascular diseases [20], cancer [21,22], ageing [23,24,25,26], bacterial and viral infections [26,27,28,29,30,31], and several neurodegenerative diseases. Indeed, evidence indicates that if HSP expression falls below a certain level, cells become sensitive to oxidative damage that accelerates ageing and protein aggregation diseases [32,33,34]. HSPs have been implicated in Parkinson’s disease, prion disease, Huntington disease, Alzheimer’s disease, amyotrophic lateral sclerosis, and polyglutamine disease, all of which have similar pathogenesis associated with protein misfolding and aggregation. In this light, HSPs are thought to prevent misfolding and aggregation of disease proteins including tau and amyloid-β in Alzheimer’s disease, α-synuclein in Parkinson’s disease, and huntingtin in Huntington disease [35]. Gezen and colleagues [36] found that high levels of HSP70 and HSP90 inhibited amyloid-β-induced neurotoxicity. Similarly, HSP70 overexpression was demonstrated to reduce α-Syn accumulation and toxicity in both mouse and Drosophila models of Parkinson’s disease [36]. In vitro studies have also portrayed that HSP70 can prevent α-Syn fibrillar assembly [37,38]. Moreover, HSP27 immunoreactivity was found to be higher in patients with amyotic lateral sclerosis, a disease characterized by progressive loss of motor neurons [39]. Notwithstanding, persistently enhanced levels of HSP can lead to inflammatory and oncogenic changes [40,41,42].

Although a fundamental role of HSPs in physiological and immunological mechanisms is evident [43], the clinical significance of HSPs remains to be further elucidated. The most commonly used methods for assessing HSPs in cells are the flow cytometric and Western blotting techniques [44,45]. However, these techniques are time consuming and therefore not adequate for the processing of a large number of samples. Moreover, flow cytometry cannot be used to detect HSPs in fluids—such as plasma and serum—which are ideal media for clinical studies. To address these problems, we have developed a single molecule assay (SIMOA) for HSP determination in cell lysates and in blood samples.

## 2. Results

In the present study a SIMOA technology for HSP detection was developed. The assay is performed in three steps: First, samples are mixed with HSP antibody capture beads for 35 min. Then, biotinylated HSP-specific antibody is added and incubated for 30 min. This is followed by a third incubation with streptavidin-β-galactosidase for 5 min and finally with resorufin β-D-galactopyranoside.

HSP-specific monoclonal antibodies (SPA-810 and SPA-800 for HSP70 and HSP27, respectively) were conjugated to Homebrew carboxylated paramagnetic beads (Quanterix Corporation, Cambridge, MA, USA) following a standard coupling chemistry, based on 1-ethyl-3-(3-dimethylaminopropyl) carbodiimide, according to the manufacturer’s protocol (Quanterix Corporation, Cambridge, MA, USA). Briefly, the monoclonal antibodies were buffer exchanged into 50 mM 2-(N-morpholino) ethanesulfonic acid (bead conjugate buffer, Quanterix Corporation, Cambridge, MA, USA) using Amicon^®^ Ultra 0.5-mL centrifugal filter devices (EMD Millipore, Billerica, MA, USA). After that, the beads were activated with 0.5 mg/mL freshly prepared 1-ethyl-3-(3-dimethylaminopropyl) carbodiimide for 30 min. The activated beads were then conjugated with appropriate concentration of HSP-specific antibodies for 2 h, and blocked with phosphate-buffered saline (PBS) containing 1% bovine serum albumin for 30 min. On the other hand, to biotinylate the HSP-specific detection antibody (SPA-812 and ADI-SPA-803 for HSP70 and HSP27, respectively), a buffer exchange into PBS on Amicon filter (EMD Millipore, Billerica, MA, USA) was first carried out and then the antibody was treated with NHS-PEG4-Biotin (Thermo Fisher Scientific, Rockford, IL, USA) at an appropriate molar ratio of biotin to antibody for 30 min. After biotinylation, the antibody was purified with PBS using Amicon filters.

Based on dose response, background level, and signal-to-background ratios, an optimum concentration of 0.5 mg/mL was considered for the capture bead and a 40-fold molar ratio of biotin to antibody was adopted for the detection antibody. Thereafter, the optimum level of streptavidin-β-galactosidase enzyme was evaluated (160 pM), applying the optimum conditions of the capture bead and detection antibody. Table 1 and Table 2 show the assay calibration performance summary using optimum conditions for HSP70 and HSP27, respectively.

### 2.1. Assay Limit of Detection

To evaluate the assay, human recombinant HSPs were used to generate standard curves under optimized conditions—by a serial dilution into calibration diluent at 8 points including blank (0–1000 ng/mL for HSP27 and 0–20,000 ng/mL for HSP70). The mean of average enzymes per bead, standard deviation (SD), and percentage coefficient of variation (CV%) were calculated for each calibrator level and the blank. The limit of detection (LOD) of the assay was estimated as blank + 2SD, and the average LOD of the ten curves was 0.16 and 2 ng/mL—for HSP27 and HSP70, respectively—and was calculated by averaging the values of all blank + 2SD.

### 2.2. Intra- and Inter-Assay Precision

Controls and panels (two each) were prepared by spiking HSP70 peptide (SPP-755) into calibration diluent and plasma, respectively, at two different levels (2000 and 66.7 ng/mL). For HSP27, human recombinant HSP27 peptide (ADI-SPP-715) was spiked into calibration diluent at two different levels (500 and 125 ng/mL). The samples were stored at −80 °C until use. Intra- and inter-assay precision were determined by testing the panels (six replicates per panel) and controls (six replicates per control), repeated on four separate days. Within-run CV was calculated for each of the panels and controls (n = 6). Inter-assay precision was determined by averaging the results from different days (n = 4) and calculating the CV% for each sample. The inter- and intra-assay coefficients of variation were less than 20% in all tested conditions (see Table 3 and Supplementary Table S1).

### 2.3. Dilutional Linearity

**Figure 1:**
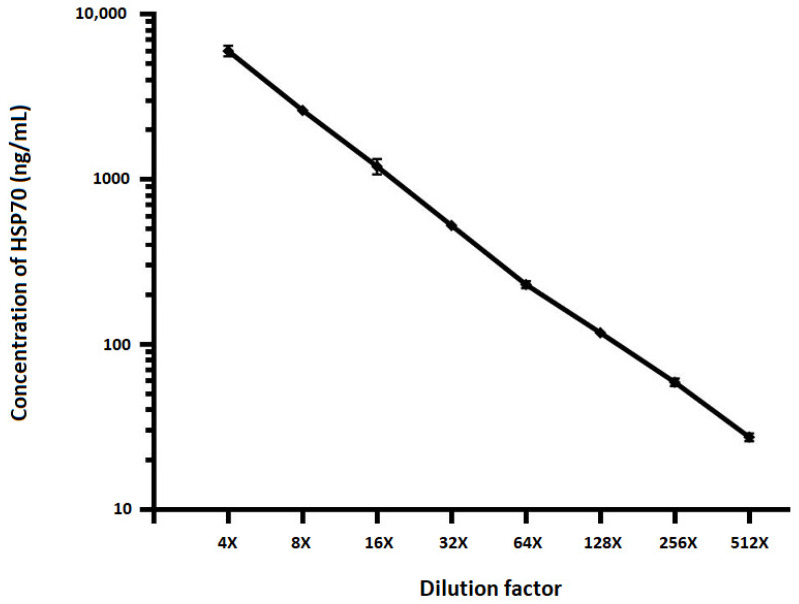
The effect of sample dilution on quantification of HSP70. To determine matrix related effects of test samples, plasma samples were spiked with recombinant HSP70 protein and twofold serial dilutions performed using sample diluent. Samples were pre-diluted four-folds and run in duplicate alongside full calibration curve. All sample concentrations were determined using the calibration curve from the run. Spearman correlation between expected and observed concentrations (R^2^ = 0.994, *p* < 0.001). The error bars are indicated.

**Figure 2:**
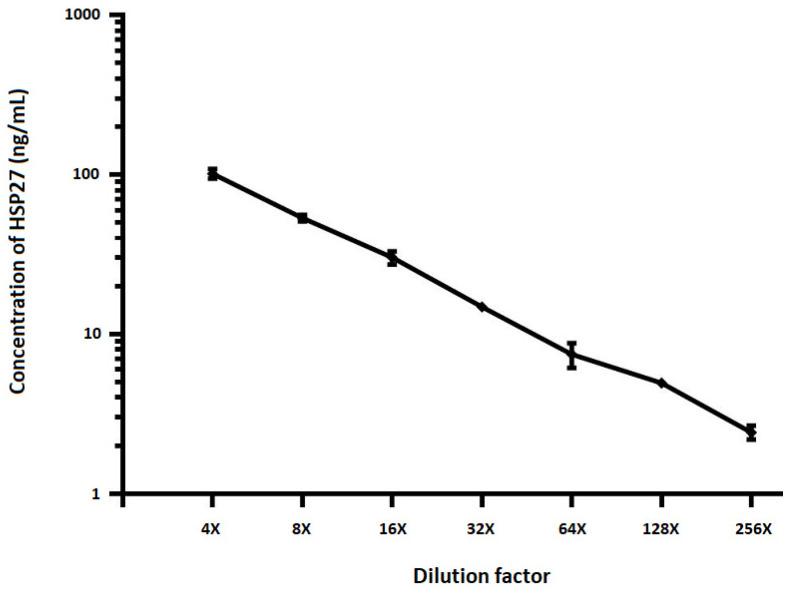
The effect of sample dilution on quantification of HSP27. To determine matrix related effects of test samples, plasma samples were spiked with recombinant HSP27 protein and twofold serial dilutions performed using sample diluent. Samples were pre-diluted four-folds and run in duplicate alongside full calibration curve. All sample concentrations were determined using the calibration curve from the run. Spearman correlation between expected and observed concentrations (R^2^ = 0.998, *p* < 0.001). The error bars are indicated.

To determine matrix related effects of test samples and minimum required dilution, a plasma sample was spiked with recombinant HSP70 (see Figure 1) and HSP27 (see Figure 2) proteins and twofold serial dilutions were performed using sample diluent. Samples were pre-diluted four-folds and run in duplicate alongside full calibration curve. All sample concentrations were determined using the calibration curve from the run. The percent recovery mean and range of recovery at each dilution was calculated using the following equation: (Concentration of diluted sample/Concentration of neat sample) × 100. The HSP levels assayed after serial dilution of samples portrayed dilutional linearity (on average 109%, R^2^ = 0.998, *p* < 0.001 for HSP27 and 93%, R^2^ = 0.994, *p* < 0.001 for HSP70).

### 2.4. Admixture Linearity

**Figure 3:**
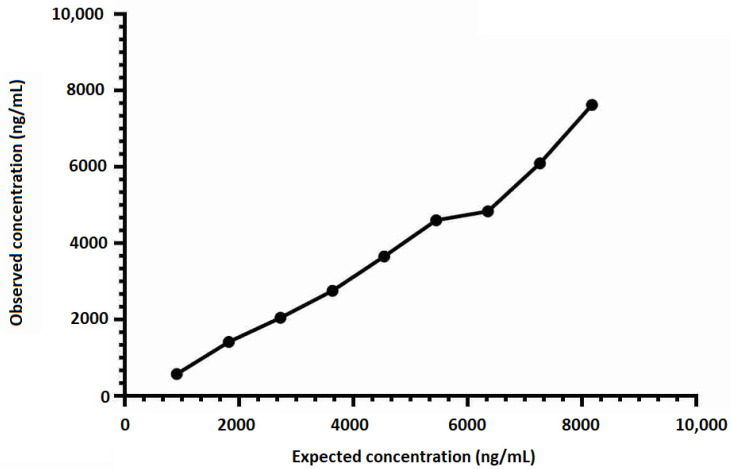
Admixture Linearity curve. To determine the linearity of sample reads along the entire calibration curve and to ensure that measurements from all parts of the calibration curve are reliable, an admixture of two plasma samples (including a sample exhibiting a low HSP70 concentration and a sample spiked with 10,000 ng/mL of human recombinant HSP70) was performed. The concentrations of the mixture were determined using the calibration curve from the run. Spearman correlation between expected and observed concentrations (R^2^ = 0.982, *p* < 0.001).

To determine the linearity of sample reads along the entire calibration curve and to ensure that measurements from all parts of the calibration curve are reliable, an admixture of two plasma samples (including a sample exhibiting a low HSP70 concentration and a sample spiked with 10,000 ng/mL of human recombinant HSP70) was performed. A high linear response was also demonstrated with admixtures of plasma samples (R^2^ = 0.982, *p* < 0.001; see Figure 3).

### 2.5. Spike Recovery

Possible interference in the determination of intracellular HSP70 concentrations by other compounds was investigated by spiking three individual plasma samples with two known concentrations of purified HSP70 and comparing the measured value with that expected. The recovered value of HSP70 in spiked plasma samples was between 57% and 95% of the expected value and was dependent on concentration and varied between samples (see Table 4).

### 2.6. Cell Lysate Samples

**Figure 4:**
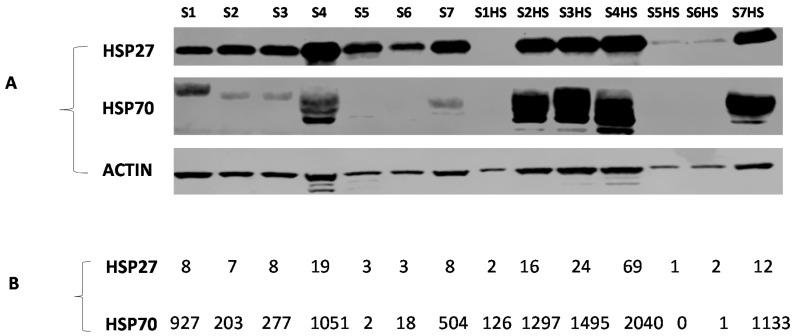
Quantification of HSP27 and 70 by Western blotting (**A**) and the newly developed SIMOA technique (**B**). Peripheral blood mononuclear cells from samples from 7 independent volunteers were lysed unheated or heat shocked at 42 °C for 1h before being lysed. S1 = sample 1 unheated; S1HS = sample 1 heat shocked; HSP = heat shock protein. For samples 1, 5 and 6, the water in the water bath—where samples were heat shocked—penetrated the Petri dishes. However, we decided not to discard them, but rather to use them—for comparison purpose—in both the SIMOA and Western blotting techniques. The signal intensity of HSP—relative to actin—obtained using Western blotting correlated significantly with HSP values using the present SIMOA technique: Spearman correlation test (r = 0.815, *p* < 0.001 and r = 0.895, *p* < 0.001 for HSP70 and HSP27, respectively).

In order to evaluate the utility of the assays for HSP27 and HSP70 measurement, peripheral blood mononuclear cells from samples from 7 independent volunteers were prepared and lysed immediately or heat shocked at 42 °C—a well-known inducer of HSPs—before lysis. As expected, the intracellular levels of HSP70 increased significantly after heat shock (*p* < 0.001). For samples 1, 5, and 6, the water in the water bath—where samples were heat shocked—penetrated the Petri dishes. However, we decided not to discard them, but rather to use them—for comparison purpose—in both the SIMOA and Western blotting techniques (see Figure 4). In addition, the levels of HSP27 and 70 obtained with the present technique were compared with their relative values determined by Western blotting. Relative HSP values obtained using Western blotting correlated significantly with HSP values of the present SIMOA technique (r = 0.815, *p* < 0.001 and r = 0.895, *p* < 0.001 for HSP70 and HSP27, respectively), indicating that the two methods are comparable (see Figure 4 and Supplementary Figure S1).

## 3. Discussion

HSPs are highly conserved proteins that play an essential role in promoting cellular survival and maintenance of normal cell function [46]. To date, although techniques for measuring HSPs exist, these assays are limited for use in specific sample types or are time consuming. With the increasing interest in therapeutic manipulation of HSPs for clinical trials, a need for an improved method to facilitate investigations of the clinical relevance of HSPs has emerged. Our approach offers advantages over the conventional methods. Firstly, the assay is carried out with full automation on the HD-1 Analyzer, with a hands-on time of one hour and a time-to-results of two hours, which makes it very efficient and time effective. Secondly, the assay allows the handling of 80 samples in one run, and beads with different spectral characteristics can be multiplexed with the possibility to measure more than one analyte simultaneously from a single sample. Thirdly, the assay can be used for serum, plasma, cell culture medium, and cell lysates.

The performance characteristics of the present digital approach to detect HSPs were evaluated, and the assay’s capability to quantify HSPs in samples of various types demonstrated. To obtain a high sensitivity while maintaining a dynamic range suitable for the detection of HSPs in various sample types, we optimized assay reagents, assay buffers, and conditions. Based on dose response, background level, and signal-to-background ratios, an optimum concentration of 0.5 mg/mL of HSP monoclonal antibody—used to coat the capture beads—and a 40-fold molar ratio of biotin to HSP polyclonal detection antibody, were found to generate the most favorable assay performance. Therefore, these conditions were applied to evaluate the performance characteristics of the present assay.

Our assay offers the advantage of a robust dilution linearity in the measurements of samples spiked with HSPs in the range of 4- to 512-fold dilution. This huge range of dilution linearity indicates that samples with HSP concentration highly above the calibration range can be diluted into range without affecting the precision of the assay, thus expanding the application of the assay to samples that have a much wider range of HSP levels. On the other hand, in non-linear assays, accurate measurements can be obtained only for samples whose values are within the calibration range [47]. Additionally, in the present assay, the admixture of two plasma samples—exhibiting very low and high levels of HSP70—portrayed linear results, suggesting that measurements from all parts of the calibration curve are reliable. Additionally, the dilution of samples mitigates matrix effects, which represents a hallmark in the accurate measurement of analytes by ELISA [48].

Reproducibility of the assay was evaluated by spiking a known amount of HSP protein into a sample or calibration diluent at three different levels spanning the calibration curve range. Both intra- and inter-assay coefficients of variation were less than 20% at all concentrations tested over a period of 4 days, suggesting that the assay is precise with a high reproducibility for HSP measurements. Lachno et al. [47] portrayed similar coefficients of variation for the quantification of amyloid-β peptide species in human plasma. Variability in atmospheric conditions as well as instability of samples and assay reagents could partly account for such variations.

Recovery of analyte in spiked human plasma ranged between 57% and 95% of the expected value and was dependent on concentration and varied between samples. This observation corroborates results reported by others—using the same technique—for the measurement of amyloid-β 1–42 peptide in human plasma [49]. A possible explanation for this difference in recovery in the present study could be the presence of HSP70-specific antibodies, which have been reported in human samples [43,50,51]. These antibodies might reflect a natural pre-existing autoimmune response or a previous encounter with infectious agents whose HSPs can elicit an anti-HSP response in the host [52]. In this light, the possibility that such anti-HSP antibodies might cross-react with epitopes found on the surface of HSPs, including the epitope recognized by the primary antibody used in this study cannot be precluded.

In order to evaluate the utility of the present assay for quantification of intracellular HSP27 and 70, peripheral blood mononuclear cells from samples from 7 independent volunteers were heat shocked or not at 42 °C, and the levels of HSP27 and 70 obtained with the present SIMOA technique were compared with those determined by Western blotting. Relative HSP values obtained using Western blotting correlated significantly with HSP values of the present technique, indicating that our method is reliable.

## 4. Materials and Methods

### 4.1. Reagents and Antibodies

HSP70 monoclonal antibody (SPA-810), HSP27 monoclonal antibody (ADI SPA-800), human recombinant HSP70 protein (SPP-755), polyclonal HSP70 antibody (SPA-812), recombinant human HSP27 (ADI-SPP-715), and polyclonal HSP27 antibody (ADI- SPA-803), were from Enzo Lifesciences (Antwerp, Belgium). Ficoll was from Nycomed (Oslo, Norway), Bovine serum albumin (BSA) was from Roche (Boehringer Mannheim, Germany), Fetal calf serum was from Biochrom (International Medical, Wavre, Belgium), RIPA buffer was made up of 1% nonidet P40, 1% deoxycholic acid, NaCl 150 mM, 0.1% SDS, 1% Triton X-100, phosphate inhibitors (50 mM NaF, 10 mM h-glycerophosphate, 1 mM Na_2_P_2_O_7_ 10H_2_O, 10 mM Na_3_VO_4_, 10 mM p-nitrophenylphosphate) and a cocktail of protease inhibitors (10:1000). Fix and Perm cell permeabilization kit was from IMTEC (Antwerp, Belgium). Phosphate-buffered saline (PBS) consisted of 40 mM Na 2HPO_4_ 2H_2_O, 135 mM NaCl, 3 mM KCl, and 1 mM KH_2_PO_4_.

### 4.2. Detailed Procedures

#### 4.2.1. Preparation of Paramagnetic Capture Beads

HSP-specific monoclonal antibodies (SPA-810 and SPA-800 for HSP70 and HSP27, respectively) were conjugated to Homebrew carboxylated paramagnetic beads (Quanterix Corporation, Cambridge, MA, USA) following a standard coupling chemistry, based on 1-ethyl-3-(3-dimethylaminopropyl) carbodiimide, according to the manufacturer’s protocol (Quanterix Corporation, Cambridge, MA, USA). Briefly, the monoclonal antibodies were buffer exchanged into 50 mM 2-(N-morpholino) ethanesulfonic acid (bead conjugate buffer, Quanterix Corporation, Cambridge, MA, USA) using Amicon^®^ Ultra 0.5-mL centrifugal filter devices (EMD Millipore, Billerica, MA, USA). After that, the beads were washed three times with PBS containing 1% Tween 20 (PBS/T) and twice with bead conjugate buffer, pH 6.2. Then, the beads were activated with 0.5 mg/mL freshly prepared 1-ethyl-3-(3-dimethylaminopropyl) carbodiimide in cold bead conjugate buffer for 30 min. The activated beads were then washed with cold bead conjugate buffer and conjugated with appropriate concentration of HSP-specific antibodies in bead conjugate buffer. After 2 h, the capture beads were washed twice with PBS/T and blocked with PBS containing 1% bovine serum albumin (PBS/BSA) for 30 min. Finally, the coated and blocked beads were washed once with PBS/T, then with 50 mM Tris buffer supplemented with 1% BSA and stored in the latter at 4 °C until use.

#### 4.2.2. Biotinylation of Detection Antibody

The HSP-specific detection antibody (SPA-812 and ADI-SPA-803 for HSP70 and HSP27, respectively) was buffer exchanged into PBS on Amicon filter (EMD Millipore, Billerica, MA, USA) and then treated with NHS-PEG4-Biotin (Thermo Fisher Scientific, Rockford, IL, USA) at an appropriate molar ratio of biotin to antibody for 30 min. After biotinylation, the antibody was purified with PBS using Amicon filters and stored at 4 °C until use. All experiments were performed at room temperature.

#### 4.2.3. Assay Validation Procedures

To determine the optimum levels of reagents, beads were captured with three different concentrations of primary HSP-specific antibodies (0.7, 0.5, and 0.3 mg/mL). In addition, the detection HSP-specific antibodies were biotinylated at 40- and 60-fold molar ratios of biotin to antibody according to the manufacturer’s protocol (Quanterix Corporation, Cambridge, MA, USA). The three capture bead concentrations were separately run against each biotinylated detection antibody to establish the optimal levels of the reagents.

#### 4.2.4. Cell Preparation

Peripheral blood mononuclear cells from samples from 7 independent volunteers were recovered as described previously [53] with slight modification. Briefly, EDTA-anticoagulated blood was diluted twice with PBS and layered over lymphoprep (2/3 blood to 1/3 lymphoprep). After centrifugation for 20 min at 500× *g*, the peripheral blood mononuclear cells were removed and centrifuged again for 10 min at 100× *g*. Thereafter, the cells were washed twice in PBS/BSA at 900× g for 3 min and resuspended in RPMI 1640 supplemented with 2 mM L-glutamine, 10% FCS, 2 mM HEPES buffer, penicillin, and streptomycin. Aliquots of the cell suspension were lysed immediately at room temperature. The remaining aliquots were plated at a concentration of 3 to 10 × 10^6^ cells/mL in 35-mm diameter Petri dishes (Becton Dickinson, Erembodegem, Belgium) and incubated overnight in a 5% CO_2_ incubator at 37 °C. After that, the culture medium was refreshed and the Petri dishes were surrounded with a parafilm band and placed in a water bath (VEL, Leuven, Belgium) at 42 °C for 1 h. HEPES buffer maintained a constant pH in the culture medium. After heat shock, cells were allowed to recover for 4 h at 37 °C in a 5% CO_2_ incubator. Then, the cells were detached from the Petri dishes with 0.5 mL trypsin–EDTA, washed twice in PBS/BSA, and counted.

#### 4.2.5. Preparation of Cell Lysates

The cells were washed four times with PBS/BSA at 900× *g* for 3 min and the pellet suspended in an appropriate volume of RIPA buffer (100 μL RIPA to 500,000 cells). This was followed by up and down pipetting until the cell suspension was homogenous and no clumps were visible. Then, the cells were incubated for 30 min on ice with occasional mixing. Next, the cell extract was centrifuged at 1600× *g* for 10 min at 4 °C and the supernatant collected as cell lysate.

#### 4.2.6. SIMOA Assay for HSP Detection

**Figure 5:**
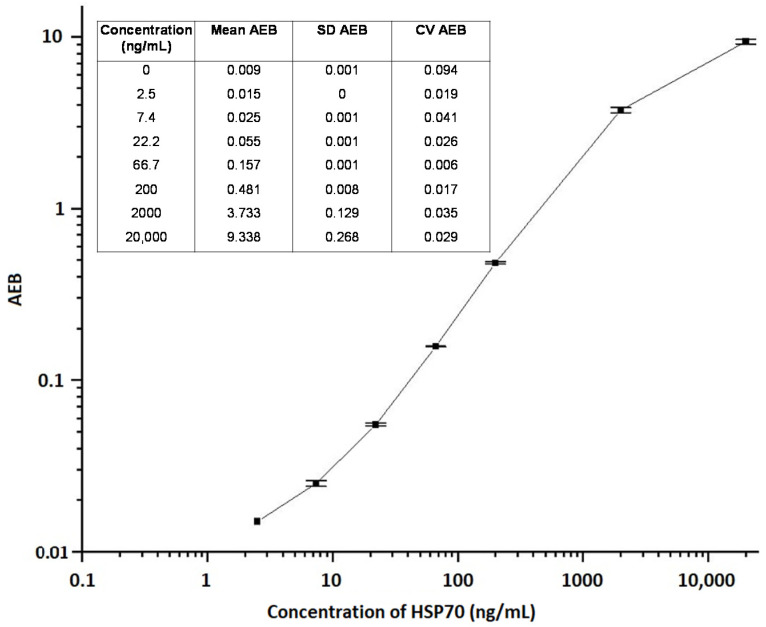
Representative standard dose-response curve and precision profile for HSP70 measurement. HSP70 was measured by quantifying signals from bound analyte on beads in units of average enzymes per bead (AEB). The average AEB (n = 2), standard deviation (SD), and coefficient of variation (CV) of each calibration point are portrayed in the embedded table. The error bars are indicated.

**Figure 6:**
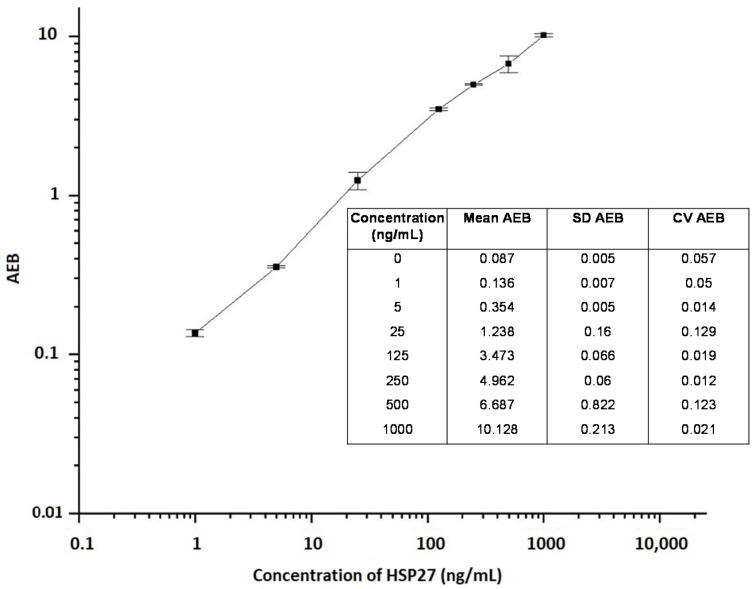
Representative standard dose-response curve and precision profile for HSP27 measurement. HSP27 was measured by quantifying signals from bound analyte on beads in units of average enzymes per bead (AEB). The average AEB (n = 2), standard deviation (SD) and coefficient of variation (CV) of each calibration point are portrayed in the embedded table. The error bars are indicated.

HSP was determined using the SIMOA technology on a fully automated HD-1 analyzer as we previously described for the detection of protein biomarker GAD65, with some modifications [54]. We chose for SIMOA because it requires less hands-on time, which makes it very suitable for the analysis of sample batches. Briefly, samples were diluted 1:4 in PBS supplemented or not with 25% fetal bovine serum, 0.5% Tween 20, and 5 mM EDTA (calibrator diluent) in Microwell™ V96 polypropylene plates (Nunc, Quanterix Corporation, Cambridge, MA, USA). Then, 100 μL of calibrators or pre-diluted samples were mixed with 35 μL of the capture bead solution (diluted 16:1000 in 50 mM Tris buffer supplemented with 1% BSA, about 3 × 10^6^/mL) for 35 min. This was followed by three washing steps with 5× PBS containing 0.1% Tween 20. After washing, 100 μL of a solution of biotinylated HSP-specific antibody (diluted 6:3333 in PBS, about 1.8 μg/mL) was added and incubated for 30 min. The plate was then washed 3 times, followed by a third incubation with 160 pM streptavidin-β-galactosidase for 5 min. After eight washes with 5× PBS containing 0.1% Tween 20, a solution of enzyme substrate, resorufin β-D-galactopyranoside (Quanterix Corporation, Cambridge, MA, USA), was added, and the beads were transferred into the SIMOA arrays for HSP detection. All incubations and washing steps were performed automatically in the SIMOA HD-1 analyzer (Quanterix Corporation, Cambridge, MA, USA) according to the SIMOA 3.0 protocol (3-step procedure) in single-analysis cuvettes. The signals from bound HSP on the beads was quantified in units of average enzyme per bead and were plotted against concentration to create a calibration curve (see Figure 5 and Figure 6; for HSP70 and 27, respectively). The HSP concentration of samples were then interpolated from the calibration curve.

#### 4.2.7. Analysis of HSP by Western Blotting

Western blotting was done according to standard procedures using specific polyclonal antibodies for HSP70 (SPA-812) and Hsp27 (ADI-SPA-803).

## 5. Conclusions

In conclusion, our SIMOA technique is reliable for the determination of HSP27 and 70 in diverse sample types. Further, the assay offers the advantage of a huge range of dilution linearity, indicating that samples with HSP concentration highly above the calibration range can be diluted into range without affecting the precision of the assay. Furthermore, the assay allows the handling of 80 samples—with a hand-on time of one hour—making it very efficient and time effective.

## Figures and Tables

**Table 1:** Assay calibration performance summary using human recombinant HSP70 for assay evaluation.

	HSP70 Concentration (ng/mL)						
Theoretical Concentration	20,000	2000	200	66.7	22.2	7.4	2.5
Assay 1	18,416	2215	185	63.6	16.9	7.2	2.0
Assay 2	19,979	1862	158	47.5	18.4	6.6	3.3
Assay 3	19,229	3034	205	73.1	18.8	7.6	2.4
Assay 4	21,281	1932	200	68.4	23.1	7.3	2.3
Assay 5	16,617	1903	217	70.7	21.7	8.4	1.3
Assay 6	17,793	2553	195	56.1	19.3	5.8	3.0
Assay 7	17,431	1981	199	54.7	20.8	6.6	1.9
Assay 8	18,432	2533	211	63.7	24.4	8.4	1.5
Inter-assay means	18,647	2251	196	62.2	20.4	7.2	2.2
SD	1487	419	18.3	8.8	2.5	0.9	0.7
CV%	8.0	18.6	9.3	14.2	12.4	12.5	31.2

Note: Eight independent experiments were carried out to evaluate the assay performance. For this purpose, eight calibration curves were generated in separate assay runs, with each calibrator run in quadruplet and the first two measurements were used to generate the calibration curve. The concentrations of the remaining 2 replicates of each standard value were determined from the standard curve and were used to calculate the inter-assay means, standard deviation (SD), and coefficient of variation (CV). HSP = heat shock protein.

**Table 2:** Assay calibration performance summary using human recombinant HSP27 for assay evaluation.

	HSP27 Concentration (ng/mL)						
Theoretical Concentration	1000	500	250	125	25	5	1
Assay 1	1107	531	260	145	44	6.4	1.3
Assay 2	1107	515	265	134	31	5.2	1.2
Assay 3	1093	469	252	147	29	5.5	1.2
Assay 4	1058	549	277	144	28	5.1	0.9
Assay 5	1083	484	262	143	27	5.4	1.1
Assay 6	1001	429	261	148	29	5.5	1.1
Assay 7	1074	584	264	125	27	5.1	0.9
Inter-assay means	1075	509	263	141	31	5.5	1.1
SD	37	52	7.5	8.4	6.0	0.5	0.2
CV%	3.4	10	2.8	5.9	20	8.3	14

Note: Seven independent experiments were carried out to evaluate the assay performance. For this purpose, seven calibration curves were generated in separate assay runs, with each calibrator run in quadruplet and the first two measurements were used to generate the calibration curve. The concentrations of the remaining 2 replicates of each standard value were determined from the standard curve and were used to calculate the inter-assay means, standard deviation (SD), and coefficient of variation (CV). HSP = heat shock protein.

**Table 3:** Assay quality control summary using human plasma and diluent spiked with recombinant HSP70.

Parameter	HSP70 Concentration (ng/mL) ^a^			
	High Control	Low Control	High Panel	Low Panel
Intra-assay means (n = 6)				
Assay 1	2359	88	1884	66
Assay 2	2174	80	1627	55
Assay 3	2435	57	1434	42
Assay 4	2061	77	2014	55
Intra-assay CV%	1	3	4	4
	7	9	7	16
	4	4	6	8
	8	4	1	18
Inter-assay means (n = 4)	2257	75	1740	55
Inter-assay CV%	8	18	15	18

Note: Controls and panels (2 each) were prepared by spiking HSP70 peptide into calibration diluent (control) and plasma (panel), at two different levels (2000 and 66.7 ng/mL). HSP = Heat shock protein, SD = standard deviation, CV% = Percentage coefficient of variation. Spiked concentration; High (2000 ng/mL), Low (66.7 ng/mL). ^a^ Concentrations were determined using the calibration curve from the runs.

**Table 4:** Recovery of spiked HSP70 from EDTA plasma.

Parameter	High (2000 ng/mL)		Low (66.7 ng/mL)	
	Measured Concentration (ng/mL)	% Recovery	Measured Concentration (ng/mL)	% Recovery
Intra-assay means (n = 3)				
Sample 1	1560	78	48	72
Sample 2	1138	57	44	66
Sample 3	1892	95	60	90

Three plasma samples were spiked with recombinant HSP70 protein at two different levels (2000 and 66.7 ng/mL).

## Supplementary Materials

The following are available online at https://www.mdpi.com/2076-0817/9/11/863/s1, Figure S1: Concentration of HSP70 and HSP27 using the newly developed SIMOA and Western blotting techniques. Table S1: Assay quality control summary using calibration diluent spiked with recombinant HSP27.
