# Supplementary material for: A Novel Bead-Based Immunoassay for the Measurement of Heat Shock Proteins 27 and 70
Source: Pathogens. 2020 Oct 22;9(11):863. doi: 10.3390/pathogens9110863 (PMC7690633; doi:10.3390/pathogens9110863)
Supplement: Supplementary file 1 [file pathogens-09-00863-s001.pdf]

A

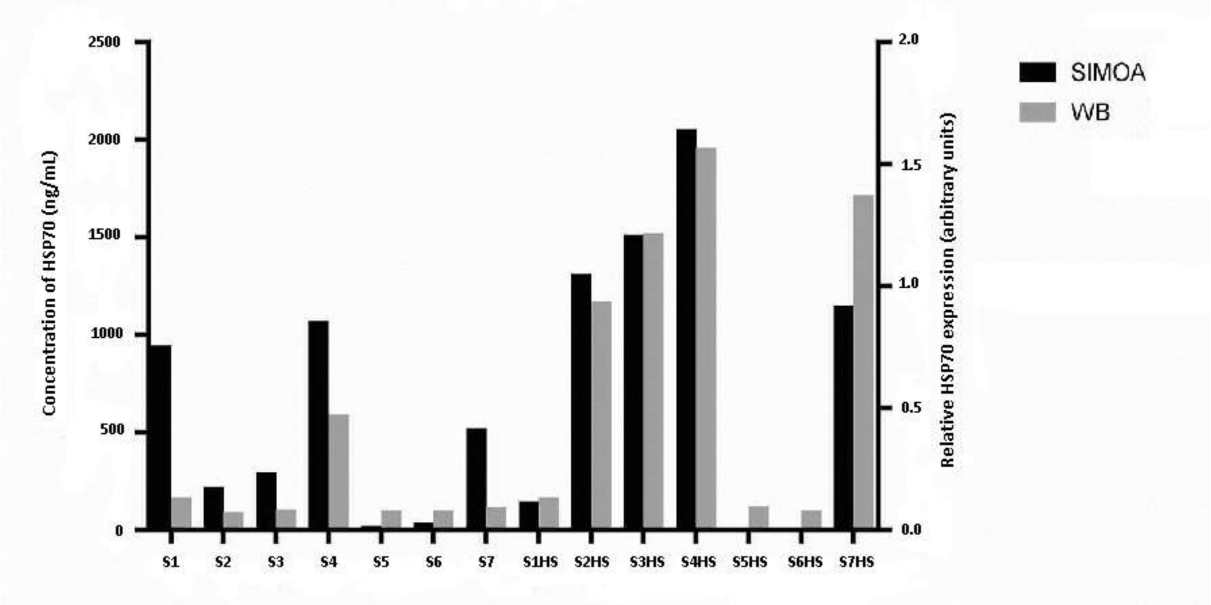

B

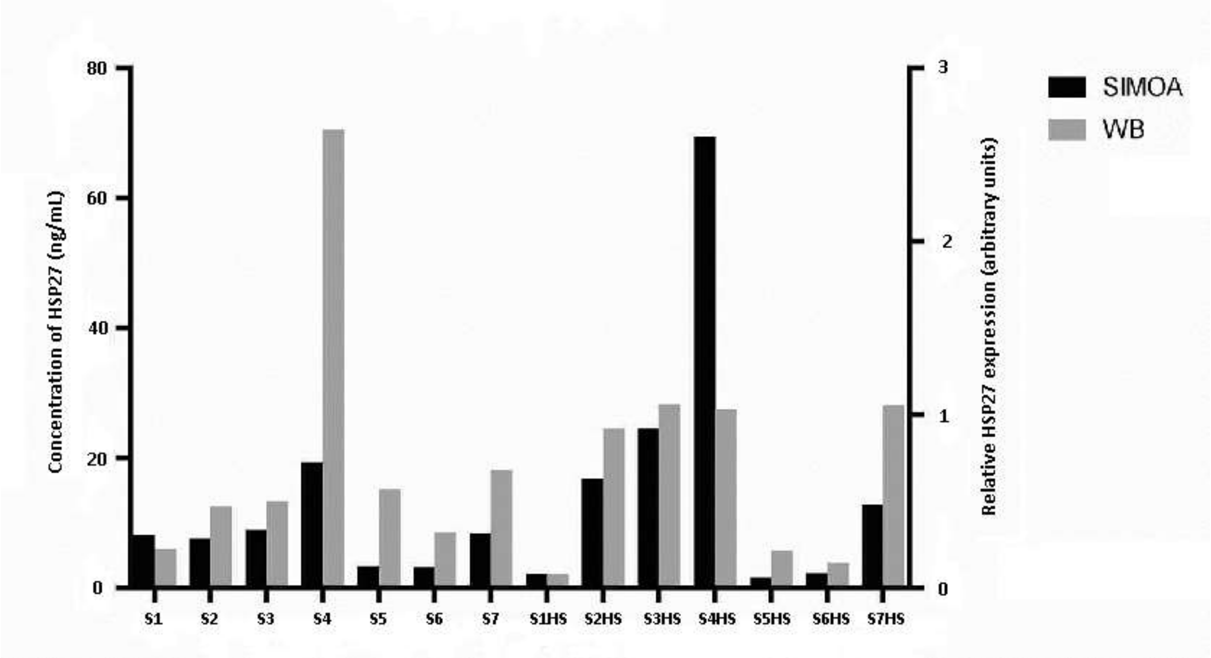

Supplementary Figure 1. Concentration of HSP70 and HSP27 using the newly developed SIMOA and Western blotting techniques.

Note. Left Y-axis: Concentration of HSP70 (A) and HSP27 (B) using the newly developed SIMOA technique. Right Y -axis: Signal intensity of Hsp70 (A) and HSP27 (B) relative to that of actin using Western blotting. S1, sample 1 unheated; S1HS, sample 1 heat shocked. The relative HSP expression obtained using Western blotting correlated significantly with HSP values using the present SIMOA technique: Spearman correlation test ($r = 0{,}815$, $p < 0.001$ and $r = 0{,}895$, $p < 0.001$ for HSP70 and HSP27, respectively).

Supplementary Table 1. Assay quality control summary using calibration diluent spiked with recombinant HSP27.

Parameter	HSP27 Concentration (ng/mL)[a]	
	High Control	Low Control
**Intra-Assay Means (n = 6)**		
Assay 1	580	160
Assay 2	599	168
Assay 3	576	132
Assay 4	555	143
Intra-assay CV%		
	6	4
	14	3
	4	13
	9	6
Inter-assay means (n = 4)	578	151
Inter-assay CV%	3	11

Note: Controls were prepared by spiking HSP27 peptide into calibration diluent at two different levels (500 and 125 ng/mL). HSP = Heat shock protein, SD = standard deviation, CV% = Percentage coefficient of variation. Spiked concentration; High (500 ng/mL), Low (125 ng/mL). [a]Concentrations were determined using the calibration curve from the runs.
